# Characterization of a non-sexual population of *Strongyloides stercoralis* with hybrid 18S rDNA haplotypes in Guangxi, Southern China

**DOI:** 10.1371/journal.pntd.0007396

**Published:** 2019-05-06

**Authors:** Siyu Zhou, Xiaoyin Fu, Pei Pei, Marek Kucka, Jing Liu, Lili Tang, Tingzheng Zhan, Shanshan He, Yingguang Frank Chan, Christian Rödelsperger, Dengyu Liu, Adrian Streit

**Affiliations:** 1 Department of Evolutionary Biology, Max-Planck-Institute for Developmental Biology, Tübingen, Germany; 2 Department of Parasitology, Guangxi Medical University, Nanning, China; 3 Friedrich Miescher Laboratory of the Max Planck Society, Tübingen, Germany; Hitit University, Faculty of Medicine, TURKEY

## Abstract

Strongyloidiasis is a much-neglected but sometimes fatal soil born helminthiasis. The causing agent, the small intestinal parasitic nematode *Strongyloides stercoralis* can reproduce sexually through the indirect/heterogonic life cycle, or asexually through the auto-infective or the direct/homogonic life cycles. Usually, among the progeny of the parasitic females both, parthenogenetic parasitic (females only) and sexual free-living (females and males) individuals, are present simultaneously. We isolated *S*. *stercoralis* from people living in a village with a high incidence of parasitic helminths, in particular liver flukes (*Clonorchis sinensis*) and hookworms, in the southern Chinese province Guangxi. We determined nuclear and mitochondrial DNA sequences of individual *S*. *stercoralis* isolated from this village and from close by hospitals and we compared these *S*. *stercoralis* among themselves and with selected published *S*. *stercoralis* from other geographic locations. For comparison, we also analyzed the hookworms present in the same location. We found that, compared to earlier studies of *S*. *stercoralis* populations in South East Asia, all *S*. *stercoralis* sampled in our study area were very closely related, suggesting a recent common source of infection for all patients. In contrast, the hookworms from the same location, while all belonging to the species *Necator americanus*, showed rather extensive genetic diversity even within host individuals. Different from earlier studies conducted in other geographic locations, almost all *S*. *stercoralis* in this study appeared heterozygous for different sequence variants of the 18S rDNA hypervariable regions (HVR) I and IV. In contrast to earlier investigations, except for three males, all *S*. *stercoralis* we isolated in this study were infective larvae, suggesting that the sampled population reproduces predominantly, if not exclusively through the clonal life cycles. Consistently, whole genome sequencing of individual worms revealed higher heterozygosity than reported earlier for likely sexual populations of *S*. *stercoralis*. Elevated heterozygosity is frequently associated with asexual clonal reproduction.

## Author summary

The vast majority of multicellular organisms reproduce sexually. Sexual reproduction is believed to be advantageous because meiotic recombination separates beneficial and deleterious mutations and generates new, possibly better allele combinations. However, sexual reproduction comes at a cost. Beneficial allele combinations are broken up and, in gonochoristic species, there is the "two-fold cost of sex" due to the investment in "unproductive" males. *Strongyloides stercoralis*, the causing agent of the grossly neglected but sometimes fatal human strongyloidiasis, appears to get the best of both worlds. Depending on the environmental conditions, *S*. *stercoralis* switches between asexual parasitic and sexual free-living generations. In the Guangxi province (China) we identified a population of *S*. *stercoralis* that appears to have recently transitioned to predominantly if not exclusively reproducing asexually. We failed to detect sexual stages and, in the genomes, we found indication of asexuality such as a high heterozygosity, compared with other populations of *S*. *stercoralis*. Additionally, global within-species phylogenetic analysis showed that in our study area, all *S*. *stercoralis* form one group of fairly close relatives, suggesting a rather recent common origin. In contrast, the hookworms (*Necator americanus*), which are phylogenetically distant from but share the infection route with *S*. *stercoralis*, in a within-species phylogenetic comparison fall into various groups, suggesting much greater diversity.

## Introduction

Soil-transmitted helminths (STHs) are parasitic worms that infect hosts by transmitting their eggs or larvae through contaminated soil. Among them are parasitic nematodes like giant roundworms (*Ascaris lumbricoides*), hookworms (*Necator americanus* and *Ancylostoma* spp.), whipworms (*Trichuris*) and threadworms (*Strongyloides*). STHs infect up to one quarter of the world's population and cause helminthiases, which are considered to be neglected tropical diseases (NTDs). Impoverished populations with limited access to clean water, sanitation, and opportunities for socioeconomic development are disproportionately affected [1]. *Strongyloides stercoralis* is the prime causative agent of human strongyloidiasis [2]. Estimates of worldwide human infection with *S*. *stercoralis* vary but go up to 370 million [3–5]. *S*. *stercoralis* is generally more prevalent in tropical and subtropical countries, and local prevalences of over 40% in particular regions have been reported [6, 7]. However, the presence of *S*. *stercoralis* and strongyloidiasis, including fatal cases, have also been reported from well-developed regions with temperate climates such as the European Union and North America [8–15].

**Fig 1:**
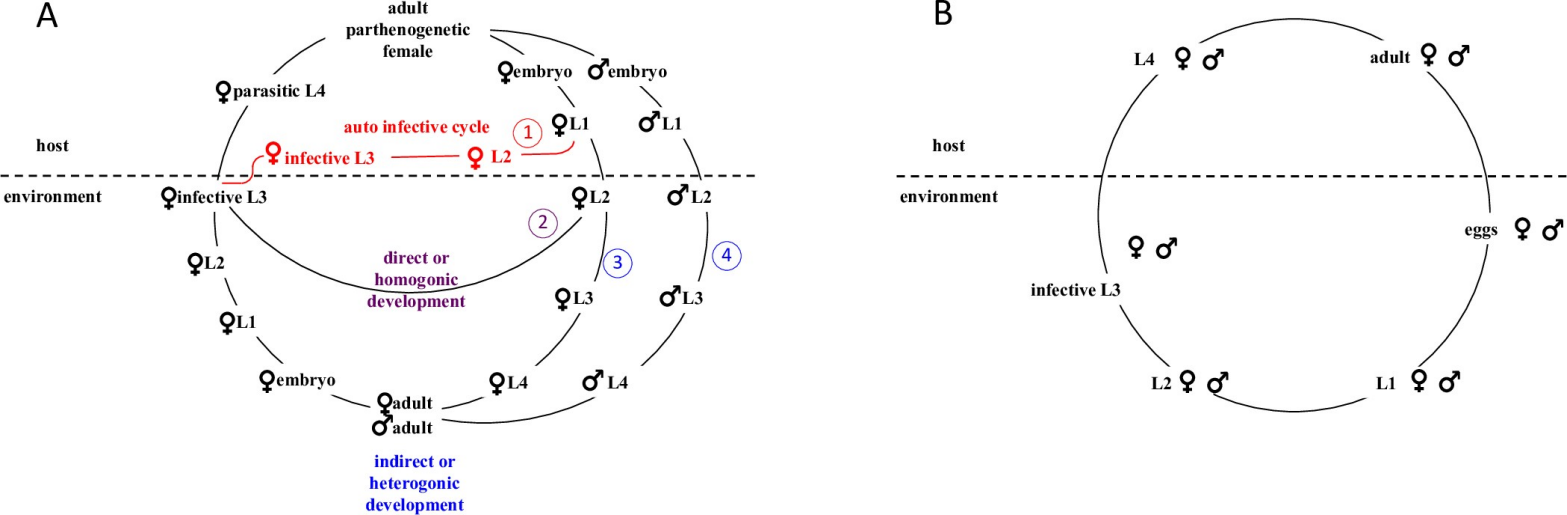
The life cycles of *S*. *stercoralis* and *N*. *americanus*. (A) The life cycle of *Strongyloides stercoralis*. The numbers refer to the numbers of the developmental options in the description of the life cycle in the text. This figure was reproduced from [50] under the creative commons license. Notice that auto infective iL3s are not normally observed in naturally deposited feces and were therefore not detectable with the methods used in this study. (B) The life cycle of *N*. *americnaus*.

*S*. *stercoralis* has a rather unique life cycle (Fig 1A) with the possibility of forming free-living adults in between parasitic generations [16–18]. The parasitic adults are all females and reproduce by mitotic parthenogenesis. Nevertheless, they can produce progeny of both sexes. Their female progeny have three developmental options. They can develop into infective third stage larvae (iL3) within the host (1 in Fig 1A) and infect the same host without ever leaving the host (auto infective cycle, notice that auto infective iL3s are not normally present in naturally deposited feces and are therefore not detectable with the methods employed in this study). Alternatively, the female larvae can leave the host with the feces as early (first stage) larvae and continue their development in the environment. There, in approximately one to two days, they either become iL3s (2 in Fig 1A), which search for another host (direct/homogonic cycle) or develop into free-living adults (3 in Fig 1A), as do all the males (4 in Fig 1A) (indirect/heterogonic cycle). The free-living adults reproduce sexually. Their progeny are all females and invariably develop into iL3s. The auto infective cycle appears to be specific for *S*. *stercoralis* and it is a prerequisite for the severe pathogenicity caused by this species [16]. This explains why strongyloidiasis in humans is a severe disease but *Strongyloides* spp. are of only very minor veterinary concern [4, 19], with the exception of great apes in captivity [20] and dogs [21], which are also hosts for *S*. *stercoralis*. The auto infective cycle allows the infection to persist in one host for many years, which is much longer than the life expectancy of an individual worm. Most of the time, infection with *S*. *stercoralis* is asymptomatic in healthy hosts, rendering it rather unlikely to be detected. If the host becomes immuno-deficient, the control of the infection may fail, resulting in a self-enhancing progression of strongyloidiasis, known as hyperinfection syndrome and disseminated strongyloidiasis, which is lethal if not treated [3–5, 22].

All species of *Strongyloides* investigated so far may undergo homogonic or heterogonic development. The switch between the two life cycles is influenced by various environmental factors, such as the immune status of the host, temperature or food availability, but also by genetic pre-disposition [23], such that different isolates may show very different homogonic to heterogonic ratios even under standard laboratory conditions [24].

Hookworms are among the most prevalent parasitic nematodes in humans. The estimation of hookworm human infection is between 576–740 million (estimate of the CDC, https://www.cdc.gov/parasites/hookworm/index.html, assessed January 21st 2019) [25]. *Necator americanus* and *Ancylostoma duodenale* are the most common human hookworm species but an increasing number of presumably zoonotic infections with *Ancylostoma ceylanicum* has been reported from Asia [26]. In China, all these three species are present. Infections with a small number of hookworms are normally asymptomatic, while more severe infections cause medical problems associated with the blood sucking life style of these worms. The life cycle of hookworm is rather simple (Fig 1B). The female and male parasitic adults mate inside the host, producing eggs which are passed by defecation. The larvae hatch and develop into iL3s in the environment and are then ready to infect the next host [27].

Hookworms and *Strongyloides* spp. are phylogenetically rather distant from each other, belonging to different major clades and their parasitic life styles have presumably arisen independently in evolution [28–30]. Nevertheless, their modes of transmission are very similar. The iL3s of hookworms and *Strongyloides* mature in the environment, penetrate the skin of the host and undergo a similar body migration. Therefore, hookworm and *Strongyloides* co-infection were frequently observed all around the world (Argentina [31], Brazil [32], Cambodia [33], Tanzania [34], Côte d’Ivoire [35], Ghana [36], Thailand [37]).

Parts of the ribosomal *cox1* gene and the nuclear small ribosomal subunit rDNA (18S rDNA, *SSU*) sequence, in particular the hyper variable regions I and IV (HVR-I and HVR-IV), are frequently used as markers for molecular taxonomy in nematodes (e.g. [29, 38–45]) including *Strongyloides* spp. [46–53]. In *S*. *stercoralis* isolated from humans the HVR-IV appears virtually invariable. Only one instance of a single nucleotide difference to the reference sequence AF279916 has been reported [49] (accession number M84229). It should, however be noted that [50] found a different HVR-IV sequence in a large portion of the *Strongyloides* sp. isolated from dogs, which are generally also considered to be *S*. *stercoralis*. In HVR-I, several sequence variants were found by multiple authors [47, 50, 51, 53]. In three recent studies, by genotyping individual *S*. *stercoralis* isolated from humans in Cambodia [50, 53] and Myanmar and Japan [51], two polymorphic positions were identified in the region around the HVR-I of human derived *S*. *stercoralis*. One is a sequence of four or five consecutive Ts located in the HVR-I proper corresponding to position number 176–179 of the reference sequence AF279916, the other one is an A/T polymorphism at position 458. Interestingly, although all three studies found worms of different haplotypes to occur sympatrically, sometimes even within the same host individual, [53] and [50] found no and [51] only very few hybrids between different haplotypes, sparking the question if multiple, genetically isolated populations of *S*. *stercoralis* exist in humans. Whole genome analysis of individual worms suggested that substantial genetic diversity exists among *S*. *stercoralis* isolated from human hosts [51, 54] but did not support the hypothesis of separate populations defined by the different *SSU* HVR-I haplotypes [50].

To differentiate the different hookworm species *Necator* spp. and *Ancylostoma* spp., the commonly used nuclear molecular markers are ribosomal ITS rather than 18S sequences [40, 41, 55–58]. Based on ITS and mitochondrial *cox1* sequences, [40, 41] defined multiple genetically separated groups of what would generally be considered *N*. *americanus* in humans in Africa and proposed that they should possibly be considered different species, i.e. *N*. *americanus* and *N*. *gorillae*.

Here we describe the isolation and genomic characterization of a population of *S*. *stercoralis* from a village and local clinics in the Guangxi province in Southern China. In contrast to earlier studies, the vast majority of individuals in this population appear heterozygous for different *SSU* haplotypes and they reproduce predominantly, if not exclusively, through the non-sexual auto-infective and homogonic life cycles. Consistent with asexual reproduction, we found elevated heterozygosity in the genomes of individual worms, compared with individuals from other populations of *S*. *stercoralis*. All *S*. *stercoralis* isolated in Guangxi were closely related arguing for a rather recent common source of infection. As a comparison, we also analyzed the hookworms isolated from the same village. While all these hookworms belonged to the species *Necator americanus*, they showed a rather high genetic diversity, even within host individuals, in comparison with other *N*. *americanus* populations.

## Materials and methods

### Ethics statements

The sampling of human derived material including the procedures to obtain informed consent, was approved by the "Medical Ethics Committee" of the Guangxi Medical University (no project specific number issued). All participants were volunteers and gave informed consent. Because not all people in the study area are literate, a protocol of oral consent was used as follows. The people were informed about the study by representatives of the local Center for Disease Control (CDC). The participants, or in the case of children their parents, showed their consent in two steps. First, they registered for the study and claimed collection containers. At this step, the participants were added to a list and assigned a number such that the health care providers could later identify, inform and treat the infected people but the sample was anonymized for the scientific analysis. Second, the participants submitted the sample the next day. Also this step was voluntary and a number of people did not return samples in spite of having claimed collection containers. Participants received a small financial compensation according to local habits. All participants found to be infected with pathogens were treated with anthelminthic drugs by the local disease control and prevention center (CDC) according to the related treatment guidelines.

Experiments involving *S*. *stercoralis* culture in host animals were in accordance with the "Guiding Opinions on the Treatment of Laboratory Animals" (issued by the Ministry of Science and Technology of the people's republic of China) and the Laboratory Animal Guideline for Ethical Review of Animal Welfare (issued by the National Standard GB/T35892-2018) and were reviewed and approved by the "Animal Care and Welfare Committee" of the Guangxi Medical University (S5 File).

### Study area

Human fecal samples were collected in Long An (LA) (23°13’N 107°25’E) and Qing Xiu (QX) (22°46’N 108°39’E) in May and June 2018. Both districts are located in the region around Nanning, Guangxi province, Southern China. These two districts were chosen either because a generally high incidence of helminth parasites had been noticed in an earlier survey (LA) or because an inhabitant had recently been diagnosed with strongyloidiasis in a local hospital (QX).

### Sample collection, stool examination and worm isolation

Sample boxes were distributed to the people who agreed to participate in this study and in the following day fecal samples were collected. In both districts (LA and QX) fecal samples were collected for two consecutive days.

To identify the helminth eggs, approximately 1g fresh feces from each sample were examined by the Acid Ether Sedimentation method as described [59–61]. In brief, feces were mixed with 7 ml 19% hydrochloric acid and large debris were removed. Then 3 ml ether was added, mixed thoroughly and centrifuged at 1500 rpm for 5 min. The centrifugation resulted in four layers, which were the ether, lipid debris, hydrochloric acid, and the sediment. After removing the upper three layers, one drop of 2% iodine was added to the sediment to stain the fixed eggs, which were observed microscopically.

To isolate worms, the rest of the fecal samples were processed as described [50]. In brief, feces were mixed with sawdust and moisturized with water, then cultured at ambient temperature for 24–48 hours to allow the larvae to develop to a stage where individuals destined to become iL3s, free-living males and free-living females can unambiguously be distinguished morphologically. Then the worms were isolated with Baermann funnels. Notice that auto infective iL3s are not normally isolated with this methodology. From the positive Baermann funnels, worms were transferred individually into 10 μl water and stored at -80°C. The samples from local clinics we obtained in the form of isolated worms conserved in 70% ethanol and stored at ambient temperature. These worms were washed twice in water, and transferred individually into 10 μl water and stored at -80°C.

Fecal samples were also collected from dogs from *S*. *stercoralis* positive households with the consent and the help of the owners. The samples were taken directly from the rectums of the animals. Feces were placed on NGM agar plates and incubated for 24–48 hours at ambient temperature. Any emerging worms were directly examined and collected.

### Single worm DNA preparation

Worms stored in 10 μl water were frozen and thawed 3 times with liquid nitrogen. Then 10 μl 2X lysis buffer (20 mM Tris-HCl pH 8. 3, 100 mM KCl, 5 mM MgCl_2_, 0.9% NP-40, 0.9% Tween 20, 240 μg/ml Proteinase K) were added and incubated at 65°C for 2 hours. The worm lysates were stored at -80°C until they were transported to Tuebingen on wet ice and stored again at -80°C until analyzed.

### PCR and sequencing of the *SSU*, ITS rDNA and the mitochondrial gene *cox*1

2.5 μl of worm lysate were used as template for PCR amplification of *SSU* HVR-I, *SSU* HVR-IV, ITS and *cox*1 by using *Taq* DNA polymerase (M0267, New England BioLabs) according to the manufacture’s protocol. Cycling protocol: An initial denaturation step at 95°C for 30 sec was followed by 35 cycles of denaturation at 95°C for 20 sec, annealing for 15 sec, extension at 68°C for 90 sec and a final extension step of 5 minutes at 68°C. The primers and the respective annealing temperatures are listed in Table 1.

**Table 1:** Primers and annealing temperatures for *SSU*, ITS rDNA and *cox*1 amplifications and sequencing.

	Primer		Sequence (5’-3’)	Anneal	Product
*SSU* HVR-I (SS and HW)	Fw	RH5401	AAAGATTAAGCCATGCATG	52°C	862 bp (SS) 883 bp (HW)
	Rev	RH5402	CATTCTTGGCAAATGCTTTCG		
	Seq	RH5403 (SS) ZS6492 (SS) RH5401 (HW)	AGCTGGAATTACCGCGGCTG AAACATGAAACCGCGGAAAG AAAGATTAAGCCATGCATG		
*SSU* HVR-IV (SS and HW)	Fw	18SP4F	GCGAAAGCATTTGCCAA	57°C	712 bp (SS) 702 bp (HW)
	Rev	18SPCR	ACGGCCGGTGTGTAC		
	Seq	ZS6269 (SS) 18SP4F (HW)	GTGGTGCATGGCCGTTC GCGAAAGCATTTGCCAA		
ITS-1, ITS-2 (HW)	Fw	NC5	GTAGGTGAACCTGCGGAAGGATCATT	50°C	1095 bp
	Rev	NC2	TTAGTTTCTTTTCCTCCGCT		
	Seq	ZS6991 (ITS-1) NC2 (ITS-2)	TTAGTTTCTTTTCCTCCGCT GCTGCGTTTTTCATCGAT		
*cox*1 (SS)	Fw	ZS6985	GGTGGTTTTGGTAATTGAATG	47°C	837 bp
	Rev	ZS6986	ACCAGTYAAACCACCAATAGTAA		
	Seq	ZS6990	GGTTGATAAACTATAACAGTACC		
*cox*1 (HW)	Fw	ZS6985	GGTGGTTTTGGTAATTGAATG	47°C	963 bp
	Rev	ZS6989	TCACCACAAACTAATACCCGT		
	Seq	ZS6989	TCACCACAAACTAATACCCGT		
*SSU* (SS)	Fw	ZS6492	AAACATGAAACCGCGGAAAG	65°C	1625 bp
	Rev	ZS6495	CGACTTTTGCCCGGTTCAAA		
	Seq	RH5403 (HVR-I) ZS6269 (HVR-IV) M13F M13R	AGCTGGAATTACCGCGGCTG GTGGTGCATGGCCGTTC GTAAAACGACGGCCAG CAGGAAACAGCTATGAC		

Fw: forward primer; Rev: reverse primer; Seq: sequencing primer; SS: *S*. *stercoralis*; HW: Hookworm.

Sequencing reactions were done with 1 μl of the PCR products and the sequencing primers listed in Table 1 using the BigDye Terminator v3.1 Cycle sequencing Kit (Applied Biosystems) according to the manufacturer’s protocol. The reactions were submitted to the in-house sequencing facility at the Max Planck Institute for Developmental Biology at Tuebingen for electrophoresis and base calling.

### rDNA and *cox1* haplotype determination and phylogenetic analysis

The sequences were analyzed with SeqMan Pro version 12 (Lasergene package; DNAStar, Inc., Madison, WI USA) and inspected manually. For position numbering the following sequences were used as reference: AF279916 for *S*. *stercoralis SSU* sequences; LC050212 for *S*. *stercoralis cox1* sequences; AJ920348 and AF217891 for hookworm *SSU* and ITS, respectively; AJ417719 for hookworm *cox1*. For *S*. *stercoralis cox1* the same 552 bp as in [50] were considered. For hookworm *cox1* the same 670 bp as in [40] were considered.

**Fig 2:**
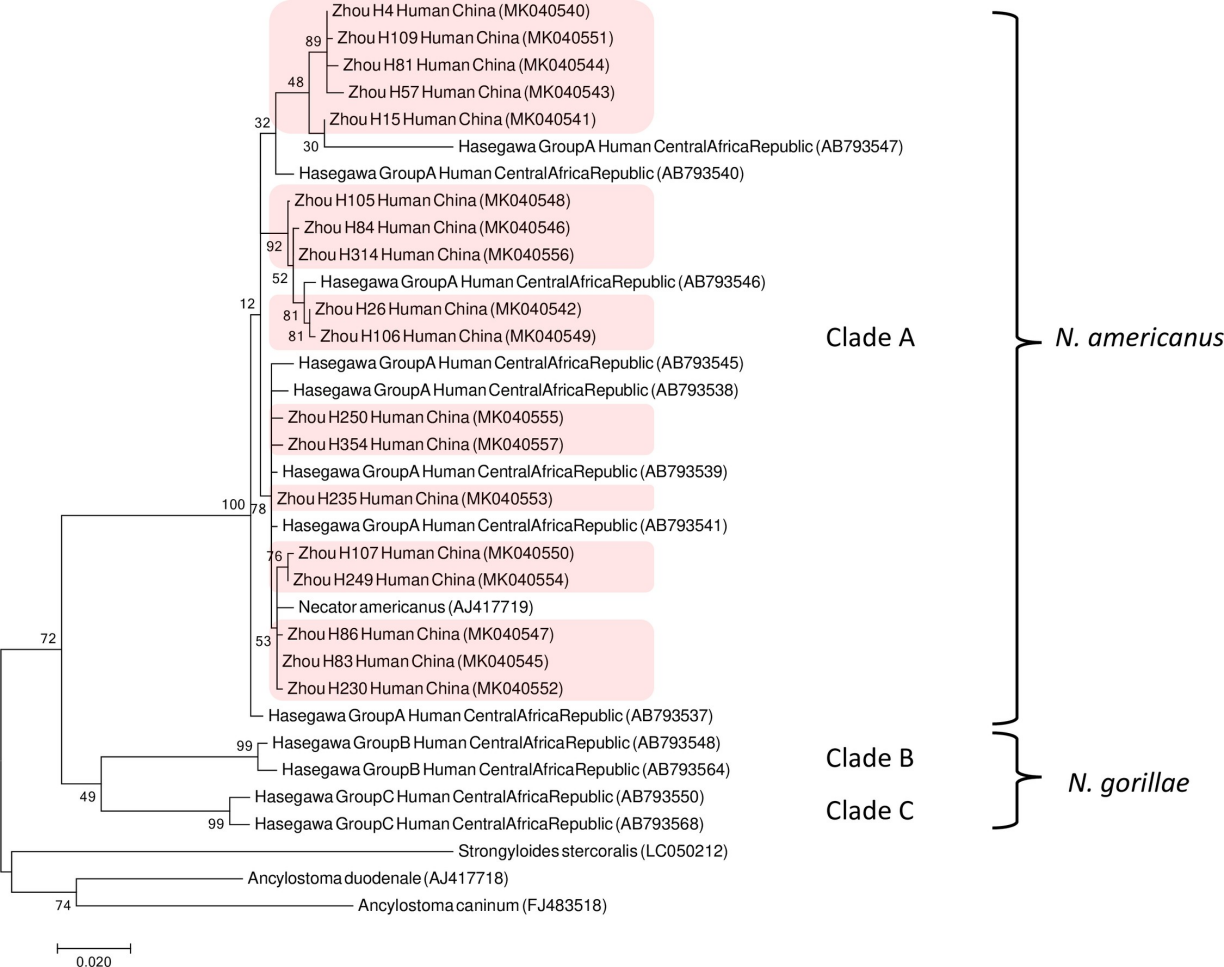
*Cox1* gene tree of different hookworm isolates. Maximum likelihood tree based on 670 bp of the mitochondrial *cox1* gene of the hookworms isolated in this study and selected published sequences. The reference sequence AJ417719 originates from the Zhejiang Province, China [72]. The scale bar represents 0.02 substitutions per site. The boot strap values represent 1000 boot strapping repetitions. The labels are composed as follows: [author of the reference] [haplotypes names according to this reference] [host the isolate was derived from] [country the isolate was isolated from] (accession number). Samples newly isolated in this study are underlaid in red. The clade and species nomenclature on the right is according to [40].

**Fig 3:**
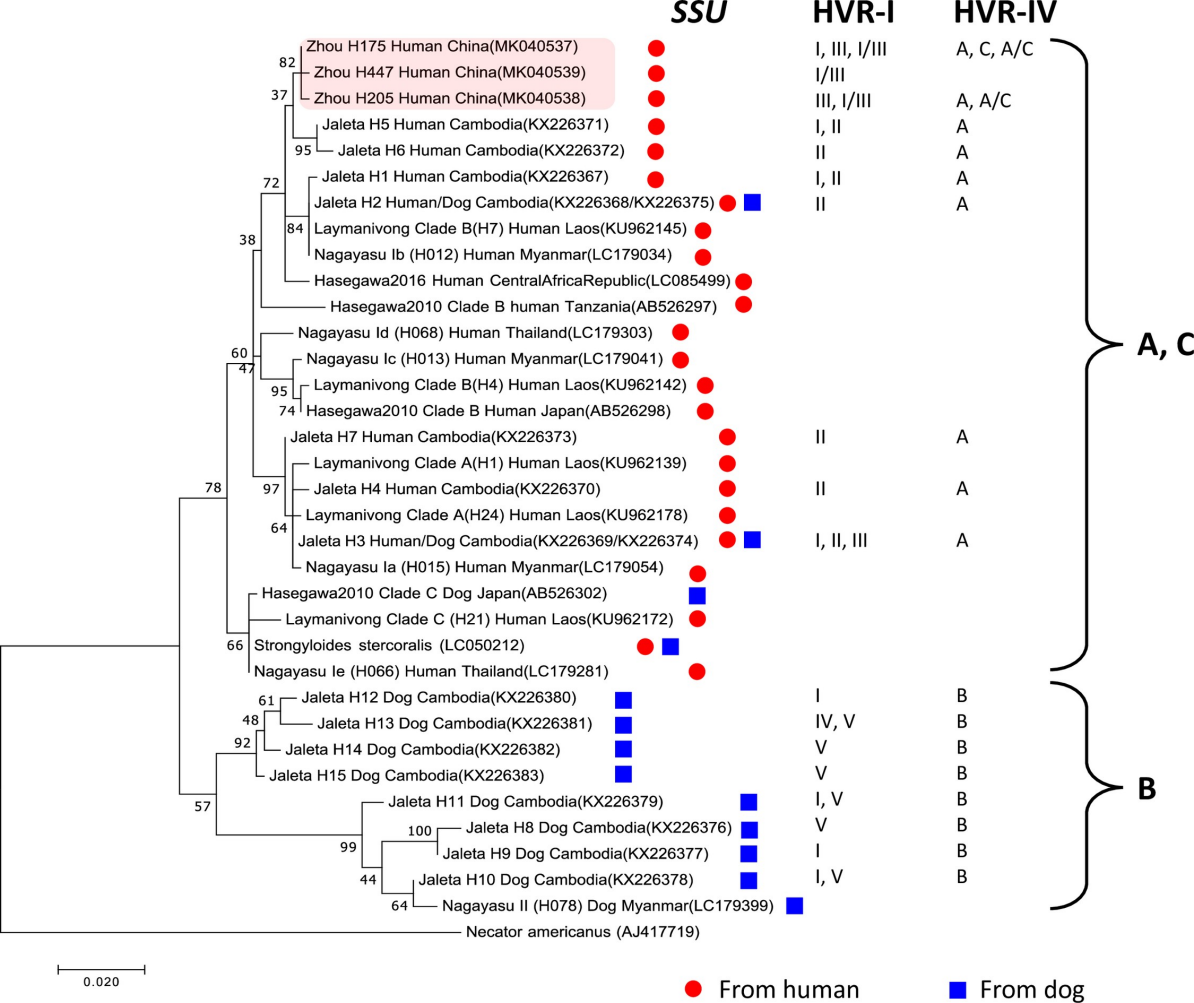
*Cox1* gene tree of different *S*. *stercoralis* isolates. Maximum likelihood tree based on 552 bp of the mitochondrial *cox1* gene. Shown are the three newly identified (red box) and selected published *S*. *stercoralis* haplotypes representing the major phylogenetic groups described in recent *S*. *stercoralis cox1* phylogenies [6, 49, 50, 73]. The scale bar represents 0.02 substitutions per site. The boot strap values represent 1000 boot strapping repetitions. The labels are composed as follows: [author of the reference] [haplotypes names according to this reference] [host the isolate was derived from] [country the isolate was isolated from] (accession number). The two columns on the right indicate the *SSU* HVR-I and HVR-IV haplotypes found among the worm individuals of the respective *cox1* haplotype.

The *cox1* sequences were aligned and phylogenetic analysis was performed using MEGA7 [62] with the maximum-likelihood (ML) model as described previously [50]. For the *S*. *stercoralis* tree, *Necator amercanus* (AJ417719) was used as outgroup species. For the *Necator amercanus* tree, *Ancylostoma duodenale* (AJ417718), *Ancylostoma caninum* (FJ483518) and *S*. *stercoralis* (LC050212) were used as outgroup species. For comparison, selected published *cox1* sequences were also included in the analysis. The corresponding accession numbers and references are listed in Figs 2 and 3.

### Cloning of the *SSU* fragments containing HVR-I and HVR-IV of individual *S*. *stercoralis*

In order to determine which HVR -I and HVR-IV haplotypes occurred in the same allele of the *SSU* heterozygous worms, we amplified 1625 bp out of 1703 bp of the *SSU* (positions 39 to 1663 in the reference AJ417719), including both, HVR-I and HVR-IV of individual *S*. *stercoralis*. 2.5 μl of worm lysate were used as template for PCR amplification by using Q5 Hot Start high-fidelity DNA polymerase (M0493, New England BioLabs) according to the manufacture’s protocol. Cycling protocol: An initial denaturation step at 98°C for 30 sec was followed by 35 cycles of denaturation at 98°C for 10 sec, annealing for 15 sec, extension at 72°C for 90 sec and a final extension step of 2 minutes at 72°C. The primers and the annealing temperature are listed in Table 1.

The PCR products were purified with Wizard SV Gel and PCR Clean-Up System (A9282, Promega). 3’ A-overhangs were added and cloned into pCR 2.1-TOPO vector and transfected into TOP10 competent *E*. *coli* cells by using the TOPO TA Cloning Kit (45–0641, Invitrogen) following the manufacturers protocol. For each *S*. *stercoralis*, multiple colonies were selected and cultured overnight at 37°C and 200 rpm in 2 ml LB medium containing 50 μg/ml ampicillin. Plasmids were isolated using the QIAprep Spin Miniprep Kit (27106, QIAGEN). The presence of an insert was confirmed by *Eco*R I (FD0274, Thermo Fisher Scientific) restriction analysis. Sequencing was done using the BigDye Terminator v3.1 Cycle sequencing Kit (Applied Biosystems) as described above with 1 μl of plasmid DNA as template and the sequencing primers described in Table 1. The sequences were analyzed as described above.

### Infection of *S*. *stercoralis* in gerbils

4-week-old female gerbils were brought from Zhejiang Medical College and injected with prednisolone acetate (3 mg) 2 days before infection and once per week after infection. Around 300 infective larvae isolated from one human host (QX24) were washed three times in tap water and incubated in PBS with antibiotic (50 mg/L streptomycin and 50 mg/L penicillin) for 1h at ambient temperature. Infective larvae were then washed again in water, and injected subcutaneously at the neck of one gerbil. Feces of the gerbil were collected daily for 1 month starting from 7-day post infection. Feces were moisturized with water and incubate at ambient temperature for 1 day followed by Baermann analysis.

### Whole genome sequencing of individual *S*. *stercoralis*

Genomic libraries of 29 *S*. *stercoralis* (26 infective larvae, 3 FL males) isolated from China and 7 *S*. *stercoralis* (5 FL males, 2 FL females) isolated from Cambodia were prepared as follows: 12 μl worm lysate were mixed with 4 μl paramagnetic bead-immobilized *Tn5* transposomes (*Tn5* expressed and purified according to [63]) and 4 μl 5X TAPS-DMF MgCl_2_ buffer (50 mM TAPS, 25 mM MgCl_2_, 50% DMF). The mixture was incubated for 14 min at 55°C. Following tagmentation, *Tn5* was stripped from DNA using SDS-containing buffer (20 μl, 30 mM Tris pH 8.0, 50 mM NaCl, 0.1% Tween 20, 0.6% SDS) with incubation at 55°C for 4 min. The beads were then washed twice with 125 μl wash buffer (30 mM Tris pH 8.0, 50 mM NaCl, 0.1% Tween 20) on a magnetic stand. PCR amplification, adapter extension and barcoding were done by adding 10 μl 5x Q5 reaction buffer, 1 μl 10 mM dNTP, 1.5 μl of 10 μM Nextera i5 primer and i7 primer, 0.5 μl Q5 high-fidelity DNA polymerase (M0491, New England BioLabs) and 35.5 μl H_2_O followed by the thermocycling program: 72°C for 5 min, 98°C for 30 sec, followed by 16 cycles of denaturation (98°C for 15 sec), annealing (66°C for 20 sec), extension (72°C for 90 sec) and cooling to 4°C. The 250–550 bp fragments of PCR products were selected with HighPrep beads (MagBio Genomics). Libraries were sequenced on an Illumina HiSeq 3000 instrument (150 bp paired-end) at the Genome Core facility at the MPI for Developmental Biology.

### Analysis of the whole genome data

#### Alignment

Raw reads were mapped to the *S*. *stercoralis* reference genome (version PRJEB528.WBPS11) using bwa mem with default settings [64]. In addition to the 36 individual *S*. *stercoralis* sequenced in this study, for comparison, we also included the published whole genome sequences of selected wild isolates from Cambodia [50], Myanmar, Japan [51, 54] and a laboratory reference isolate PV001 derived from USA [65]. For more details see S4 File.

#### Variants calling

Variants deviating from the *S*. *stercoralis* reference genome were called as described previously [66]. In brief, raw variants were called using the mpileup, bcftools, and vcfutils.pl programs of the SAMtools suite (version 0.1.18) [67] and filtered for variants with quality values ≥20. Heterozygous sites were extracted based on the attributes in the vcf files (AF1>0.4 & AF1<0.6). Heterozygosity was calculated as the fraction of heterozygous sites on the X chromosome and autosomes. Only samples comprising >80% of genomic regions with 15x depth were included for heterozygosity analysis. A Wilcoxon test was performed to evaluate the differences of autosomal heterozygosity between populations. The X chromosome was excluded from the statistical analysis because only females are informative and there were only two females derived from the Cambodian population.

For analysis of population structure, all variant sites were pooled and called in all samples in order to get the full genotypic data including reference alleles.

#### Population structure

The genome-wide phylogeny was computed by the neighbor-joining method as implemented in the phangorn R package [68] and is based on 1180 variant sites that were called as homozygous in all samples (see [66, 69] for further details). To look for potential evidence of recent or ancient recombination events, a subset of samples (three male samples from China, two reference samples, and four highly covered Cambodian samples) were selected to compute neighbor joining trees for the largest *S*. *stercoralis* contigs (S1 Fig).

In addition, PCA was performed based on a set of 910 SNPs (distributed across the whole genome, genotyped in all samples, including heterozygous sites, down-sampled to 1 SNP per 5kb) with the help of the smartpca program of the eigensoft package (version 5.0.1) [70].

#### Nucleotide diversity and analysis of coding sequences

Nucleotide diversity (π) was calculated as the mean fraction of nucleotide differences in pairwise comparisons within and between populations. For these estimates, we ignored heterozygous calls and assumed that 90% of the *S*. *stercoralis* genome was covered in both samples. Heterozygous non-reference alleles from the Chinese population were extracted and their frequency was quantified in the Chinese and the Cambodian populations. *S*. *stercoralis* gene annotations (version PRJEB528.WBPS11) were used to assess the effect of substitutions on the coding sequences.

## Results

### Liver fluke, hookworm and *S*. *stercoralis* are the gastrointestinal helminths detected in our study area

In the village LA, fecal samples were collected from 108 persons. We detected liver fluke (*Clonorchis sinensis*) (23 = 21.3%) and hookworms (12 = 11.1%) but no *S*. *stercoralis*. In the village QX, fecal samples were collected from 98 persons. We detected liver fluke (*C*. *sinensis*) (59 = 60.2%), hookworms (17 = 17.3%) and *S*. *stercoralis* (7 = 7.1%). For full information see S1 File. Further, we sampled seven of the eight dogs present in the three dog owning households with *S*. *stercoralis* positive people. No *S*. *stercoralis* were found in these dogs.

### The hookworms in the study area are genetically diverse

Since several species of hookworms are present in China [71] and they are not easily distinguishable by morphology, we determined the *SSU* sequences of 231 hookworms from 19 human hosts (11 from LA, 8 from QX). All of them were identical with the published sequence of *Necator americanus* (AJ920348).

In order to connect our work to [40, 41], which did not report the *SSU* sequences of its isolates, we determined the ITS-1 and ITS-2 sequences of 108 hookworms and 670 bp of the mitochondrial gene *cox1* of 100 hookworms from the 19 host individuals. All of them had the same ITS sequences (MK036418 [ITS-1] and MK036419 [ITS-2]), which correspond to ITS type I as defined by [40].

For *cox1* we identified a total of 18 different haplotypes (GenBank accession numbers MK040540-MK040557). 6 *cox1* haplotypes were present in more than one host individual and multiple *cox1* haplotypes were found in 8 of the 19 host individuals. For more details see S2 File. A phylogenetic comparison with the *cox1* haplotypes identified in earlier studies [40, 41] (Fig 2) showed that all our 18 *cox1* haplotypes fall into clade A as defined by [40]. Taken together, our molecular data indicate that all hookworms isolated in this study are *N*. *americanus* of ITS type I and *cox1* clade A sensu [40].

### The *S*. *stercoralis* in the study area are very closely related

Partial sequence (552bp) of the mitochondrial gene *cox1* was obtained from 69 *S*. *stercoralis* representing all seven positive human hosts in the village and one of the patients from a local hospital. All the 53 worms from six hosts shared the same haplotype (H175, GenBank accession number MK040537) while all the 15 worms isolated from the seventh host were of another haplotype (H205, GenBank accession number MK040538), which differed at one position from H175. In the hospital derived sample we identified a third haplotype (H447, GenBank accession number MK040539) that differed from H205 and H175 by 2 and 1 nt, respectively. For more details see S3 File. To examine the phylogenetic relationships, we reconstructed a maximum-likelihood tree with our and selected published *cox1* sequences [6, 48–51] (Fig 3). Our *cox1* haplotypes group clearly with the ones found in Cambodia in humans and dogs but not with the dog specific ones [50]. With moderate bootstrap support, these haplotypes could be assigned to a group described as clade B by [6] or clade Ib by [51]. Our attempt to culture this isolate of *S*. *stercoralis* in gerbils failed.

### Most *S*. *stercoralis* in the study area are hybrids for different *SSU* haplotypes

A total of 177 *S*. *stercoralis* from 9 humans were sequenced at the *SSU* HVR-I and/or HVR-IV loci. Only 2 infective larvae and 3 free-living males appeared homozygous or hemizygous (the *SSU* is on the X chromosome) for either one of the HVR-I haplotypes I and III described by [50] (Tables 2 and 3). In HVR-IV the same 5 worms plus another 28 infective larvae appeared homozygous or hemizygous for either haplotype A or C. Haplotype A is the typical haplotype for *S*. *stercoralis* isolated from humans [50]. Haplotype C is a novel haplotype identified in this study, which has a T deleted at position 1265 (compared with AF279916) (Tables 2 and 3).

**Table 2:** The *SSU* HVR-I and HVR-IV polymorphisms of *S*. *stercoralis*.

HVR-I	176 bp	458 bp	HVR-IV	1265 bp
Haplotype I	4T ATA T	T	Haplotype A	ATTTT GTTTA TTTT A-A TAT
Haplotype II	5T ATA T	T	Haplotype B	ATTT- GTTTA TTTT TTA TAT
Haplotype III	5T ATA T	A	Haplotype C	ATTT- GTTTA TTTT A-A TAT
Haplotype IV	3T ATA C	T		
Haplotype V	4T ATA C	T		
Haplotype VI	4T ATA T	A		

Haplotype nomenclature: Haplotypes I-V, A,B [50]; haplotype VI [21]; haplotype C this publication. bp indicates the positions in the reference sequence AF279916.

**Table 3:** *SSU* HVR-I and HVR-IV genotypes of individual *S*. *stercoralis*.

*SSU* Host^a^	4T/5T	-	4T/5T	4T/5T	4T/5T	-	5T	4T	Total
	T/A	-	T/A	T/A	-	-	A	T	
	A	A	A/C	-	A/C	A/C	A	C	
QX105			35	1	6	1	1 (male)		44
QX14				1	1				2
QX24			36	3	4	6		2	51
QX32	26	2							28
QX87						1		1 (male)	2
QX97			19	4	2		1 (male)		26
QX121			1						1
Patient A*					1	14			15
Patient B*				1		7			8

Individual *S*. *stercoralis* were sequenced at HVR-I and HVR-IV. Rows 1–3 represent polymorphic sequences in HVR-I (176 bp and 458 bp of AF279916) and HVR-IV (1265 bp of AF279916). Rows 4–12 represent one host individual each. Numbers indicate the number of *S*. *stercoralis* with the HVR-I/IV haplotype combinations specified in rows 1–3.

^a^The host is defined by the letter code for the village followed by the host individual number. Notice that worms from the same host may or may not be clonal siblings.

*Host from local hospital.

-: nucleotide sequence at designated position was not determined.

Interestingly, unlike in earlier studies [50, 51, 53], in this study the vast majority of *S*. *stercoralis* individuals appeared to be heterozygous at the *SSU* locus (Table 3). All of them can be explained with the hypothesis that the worms were hybrids of two haplotypes described in Table 2. To confirm this and to determine which combinations of HVR-I and HVR-IV haplotypes existed we amplified a 1625 bp fragment containing both HVRs from the individual worms described in Table 4, cloned the PCR product and sequenced individual clones.

**Table 4:** Combinations of *SSU* HVR-I and HVR-IV haplotypes.

Worm^a^	Country^b^	Sex^c^	I	I	II	II	III	III	VI	I	IV	V	Total
			A	C	A	C	A	C	A	B	B	B	
QX32(1)	Cn	F	8				6						14
QX32(2)	Cn	F	5		1		6		1				13
QX24(1)	Cn	F		3			1						4
QX24(2)	Cn	F		1			10						11
QX97(1)	Cn	F		8		1	4						13
QX97(2)	Cn	F		8		1	3	1					13
QX105(1)	Cn	F		1		1	2	2	1				7
QX105(2)	Cn	F		3			3	2					8
QX105(3)	Cn	F	1	1			16						18
DC44(1)	Kh	M			16								16
DC108(1)	Kh	M					16						16
DC79D4(1)*	Kh	M										18	18
DC86D2(1)*	Kh	M									13		13
DC79D3(1)*	Kh	M	13		5								18
DC79D3(2)*	Kh	M			16								16
KP55D2(1)*	Kh	M								24		2	26
KP55D2(2)*	Kh	M								7			7

A region spanning HVR-I and HVR-IV of individual *S*. *stercoralis* was PCR amplified and the product cloned. Individual clones were sequenced.

Rows 1 and 2 represent the HVR-I and HVR-IV haplotypes. Rows 3–19 represent one worm each. Numbers indicate the number of clones with the HVR-I/IV combination designated in rows 1 and 2.

^a^The worm is defined by the letter code for the village followed by the host individual number, and the worm individual number in brackets. Notice that worms from the same host may or may not be clonal siblings.

^b^Cn: China; Kh: Cambodia.

^c^F: female (iL3); M: male.

*Isolated from dog host.

From host QX32, where all *S*. *stercoralis* isolated from this host were hybrids in HVR-I but not in HVR-IV, the two dominant *SSU* alleles were haplotypes I and III (HVR-I) in combination with haplotype A (HVR-IV). From the other three hosts (QX24, QX97 and QX105), all *S*. *stercoralis* were hybrids in both HVR-I and HVR-IV. Here the two dominant *SSU* haplotypes were I (HVR-I) with C (HVR-IV) and III (HVR-I) with A (HVR-IV) (Table 4).

### Intra-individual variability of the *SSU* sequence was detected in *S*. *stercoralis*

In several *S*. *stercoralis* females analyzed, we identified more (up to 4) than the two *SSU* haplotypes expected for heterozygous animals (Table 4: rows 3–11). For comparison, we repeated the experiment with *S*. *stercoralis* males from Cambodia [50] (Table 4: rows 12–19). In 2 cases, we found more than one *SSU* haplotype (notice that the *SSU* is on the X chromosome). This suggests that in the population under study and, maybe to a lesser extent, in the population in Cambodia there is appreciable *SSU* sequence variation among the different rDNA copies within a haploid genome. Intra-individual variability of rDNA sequences, although apparently very rare in animals, has been observed before, for example in American sturgeons [74] or in the plant parasitic nematode *Rotylenchulus reniformis* [75]. So far, the variant 4T+A, which occurred as a minor variant and in combination with haplotype A (HVR-IV), had not been reported in any of the studies from East Asia but has recently been observed in *S*. *stercoralis* from dogs in Switzerland by [21] and named haplotype VI by these authors.

### *S*. *stercoralis* populations are geographically clustered

In order to extend our analysis beyond the *cox1* and *SSU* sequences, we sequenced the whole genome of individual *S*. *stercoralis*. For comparison, the published genome sequences of selected *S*. *stercoralis* samples from Cambodia [50], Myanmar and Japan [54] were also included in this analysis.

#### Phylogeny

**Fig 4:**
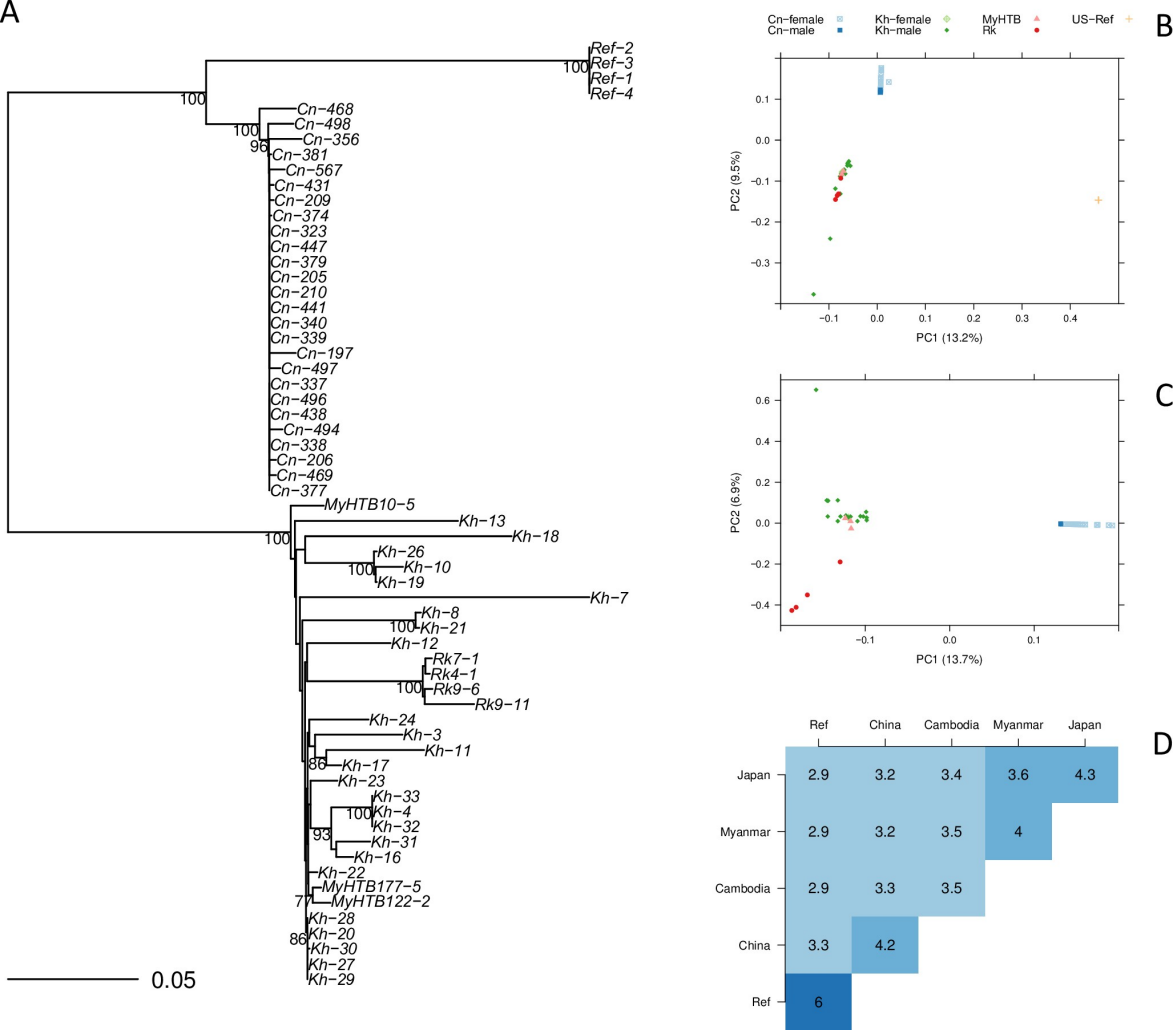
Population structure of *S*. *stercoralis*. (A) Phylogenetic relationships between the Chinese and selected *S*. *stercoralis* samples are visualized as a neighbor-joining tree that was calculated from 1180 concatenated variant sites that were genotyped as homozygous in all samples. (B) Principal component analysis was performed on 910 homozygous and heterozygous SNPs that could be genotyped in all samples. (C) Principal component analysis without the reference strain. (D) Nucleotide diversity (π) was calculated from homozygous SNPs as the mean fraction of nucleotide differences in pairwise comparisons within and between populations. The heatmap shows the negative logarithm (base 10) of π. Cn: China; Kh: Cambodia; MyHTB: Myanmar; Rk: Japan; Ref: US-derived reference laboratory strain PV001. For Cn and Kh all but three (very low read coverage) whole genome sequences determined for this study and for [50] were included. For MyHTB and Rk, we selected samples representing one outlier (Rk9-11) and the two clusters in Fig 5 of [54]. Selection within the clusters was random.

We reconstructed a phylogenetic tree based on the whole genome sequences. A clear geographical separation was observed. Samples from China, Cambodia/Myanmar, Japan and USA (reference) are grouped into different clades according to their country of origin. The only exception is that the Myanmar and the Cambodian samples show no clear separation. The Cambodian samples show higher within location diversity compared with the Chinese samples (Fig 4A).

#### Principal component analysis (PCA)

Principal component analysis (PCA) also shows a geographical clustering. All samples collected from different locations are separated from the reference strain [65] by PC1 (13.2%). Samples from China are separated from other populations by PC2 (9.5%) (Fig 4B). Since the US derived reference lab strain PV001 was very different from all the wild samples, we repeated the analysis without the reference. Hereafter, the samples from China, Cambodia and Japan are separated by PC1 (13.7%), whereas Myanmar and Cambodian samples remain mixed (Fig 4C).

#### Nucleotide diversity

Nucleotide diversity (π) within and between geographic populations is shown as a heatmap (Fig 4D). Within the population, the average distance between Cambodian samples is the highest (10^−3.5^) among all the 5 geographic populations, indicating a higher diversity among the Cambodian population. Between locations, the diversity is comparable between Cambodia, China, Myanmar and Japan (10^−3.2^–10^−3.6^). Interestingly, the samples from China appear, to a moderate extent, closer to the US derived reference strain, compared with the other samples.

**Fig 5:**
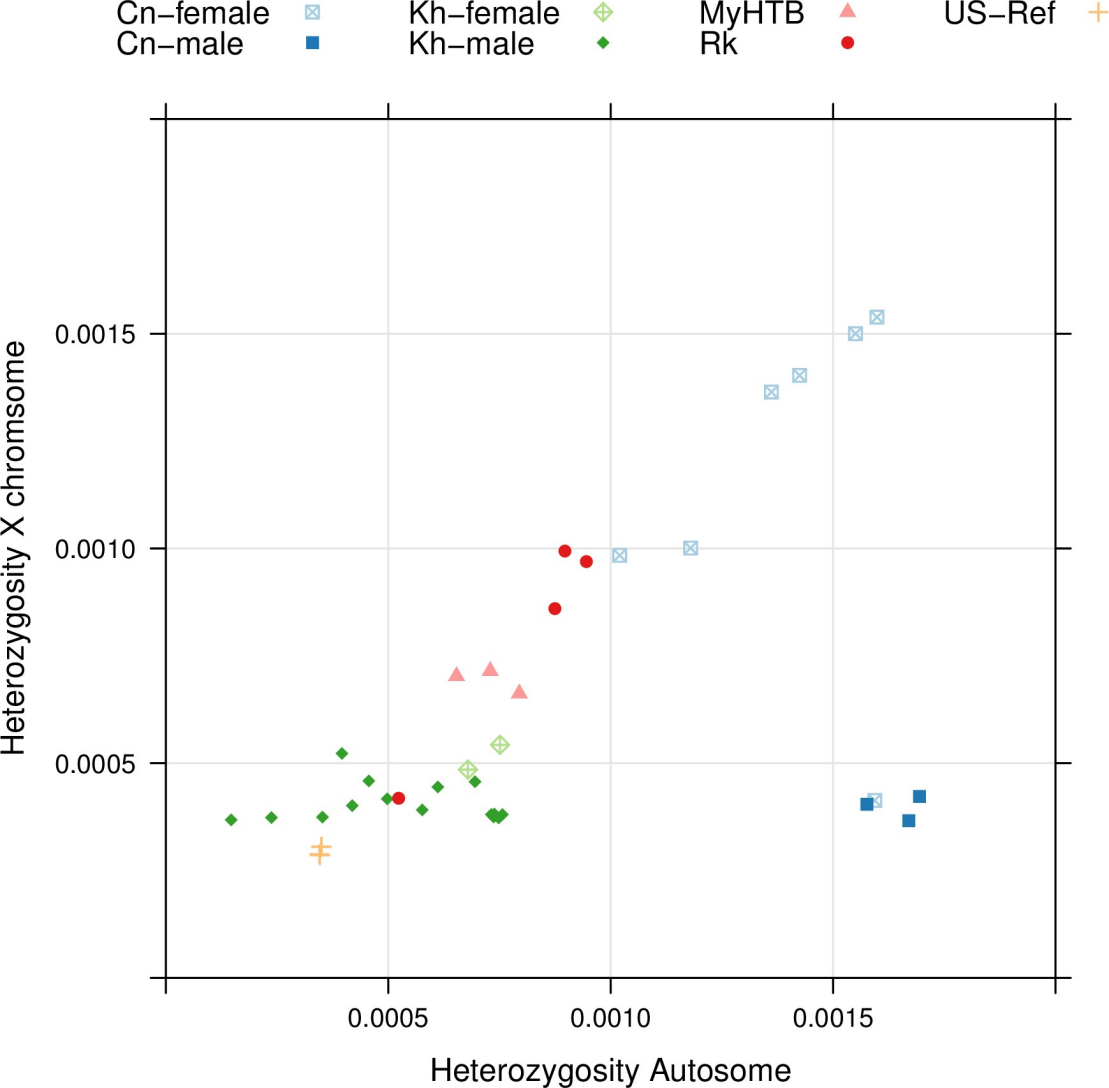
Genomic heterozygosity of individual *S*. *stercoralis*. The heterozygosity on the autosomes is plotted against the heterozygosity on the X chromosome for *S*. *stercoralis* individuals from different geographical locations. Cn: China; Kh: Cambodia; MyHTB: Myanmar; Rk: Japan; Ref: US-derived reference laboratory strain PV001. All samples in Fig 4 that fulfilled the read coverage criteria described in Materials and Methods were included in this analysis. The samples from Cn represent four different hosts from the village, two hospital patients and all three *cox1* haplotypes (c.f. S4 file).

### *S*. *stercoralis* in the study area reproduce predominantly, if not exclusively, asexually

#### Free-living adult *S*. *stercoralis* were virtually not detected

Noticeably, all (thousands) *S*. *stercoralis* we observed were infective larvae, except for 3 free-living males (isolated from 3 different human hosts). No free-living female was found. This suggests that *S*. *stercoralis* in our study area reproduces predominantly, if not exclusively asexually through the homogonic and/or the auto infective cycles. To further test this, we looked for the presence of signs of extended times of asexual reproduction in the genomes.

#### High heterozygosity across the genome

Due to the absence of meiotic recombination, in asexual organism the divergence between homologous chromosomes is expected to increase in a process known as the Meselson effect [76]. Therefore, if the Chinese population is indeed asexual, one would expect to observe a higher number of heterozygous positions compared with, for example, the samples from Cambodia, where large numbers of sexual free-living individuals were observed and sexual reproduction occurs presumably fairly frequently [50].

To detect such genomic hints for asexuality, we first compared the heterozygosity of individual *S*. *stercoralis* isolated from the different geographical locations (Fig 5). The heterozygosity on the autosomes and X chromosome were calculated separately. Theoretically, the heterozygosity on the X of males is zero because there is only one copy. The low, but not zero, measured heterozygosity of the X chromosome in males reflects the background of false positives caused for example by sequencing artifacts and errors in the reference genome assembly and variant calling. The heterozygosity in Cambodian samples is generally the lowest among the wild isolates and in the Myanmar samples it is only slightly higher. As described in [54], except for the one outlier, the heterozygosity in the Japanese sample is higher than in Myanmar, a finding these authors attributed to the extended time these worms had reproduced through the clonal auto infective cycle. The Chinese samples are even more heterozygous (Wilcoxon test: p = 2.371e-07 compared with Cambodian samples, p = 0.001998 compared with Japanese samples, autosome only).

In general, the females lay very close to the diagonal, indicating there is no difference in heterozygosity between the autosomes and the sex chromosome. The only exception is one Chinese female (Cn-323) with male-like low heterozygosity on the X chromosome (Fig 5). Interestingly, it is the only female from China in this analysis that appeared homozygous at the *SSU*. Read coverage confirmed that the individual is indeed a female with two X chromosomes (see PRJNA517237). A possible explanation is that it was the product of a rare sexual event between close relatives in the process of which the X chromosome but not the autosomes became homozygous.

#### Most variants arose prior to asexuality

Secondly, since the new mutations never become homozygous in an asexual population, it is expected that purifying selection is reduced and slightly deleterious mutations can accumulate, which is manifested in an increased proportion of nonsynonymous changes [76]. Thus, we asked if there had been an accumulation of nonsynonymous mutations in the Chinese population.

**Fig 6:**
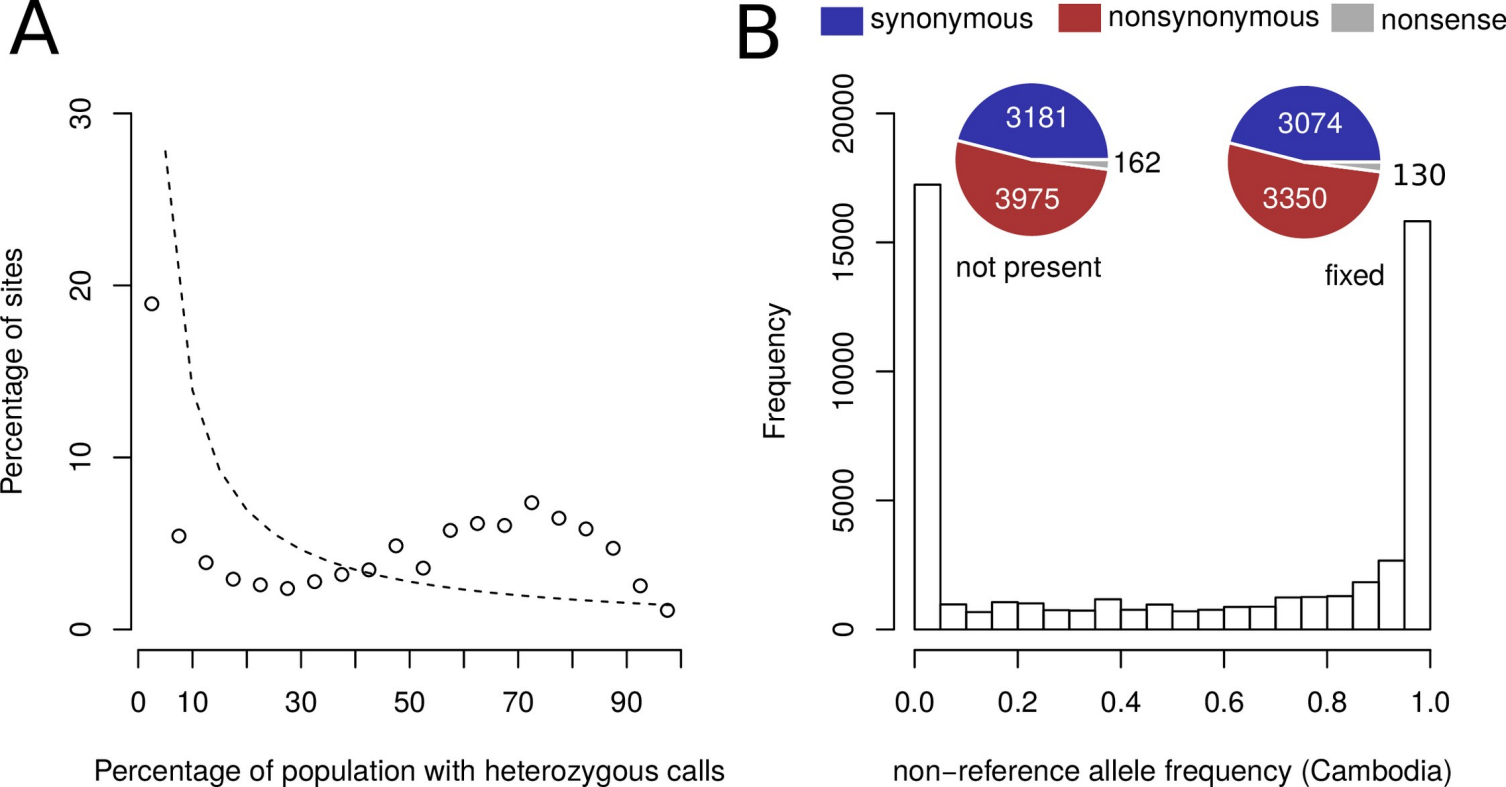
The heterozygosity was derived from ancestral polymorphisms. (A) The frequency of heterozygous calls in the Chinese population was quantified and compared to a neutral expectation of derived allele frequencies under a genetic drift model (1/k where k is the number of samples (dashed line) [66, 77]. (B) The frequency of the non-reference alleles derived from (A) was quantified in the Cambodian population. Large fractions of these alleles are fixed in Cambodian population (“fixed”) and the other large fraction of alleles is not found in Cambodian population (“not present”). The numbers of nonsynonymous variants in the “not present” and “fixed” group are shown in pie charts.

We extracted the heterozygous sites in predicted coding regions present in at least two Chinese samples and calculated the frequency of heterozygous calls in Chinese population (Fig 6A). Then we quantified the frequency of the non-reference alleles in the Cambodian samples. One large fraction of such non-reference alleles was fixed in the Cambodian population. These are old alleles shared between the Cambodian and Chinese populations (“fixed” in Fig 6B). In contrast, in the other large fraction, the reference alleles were fixed in Cambodia. These non-reference variants present in Chinese but not Cambodia population are candidates for new mutations arisen in Chinese population after the transition to asexual reproduction (“not present” in Fig 6B). We compared the ratio of nonsynonymous and synonymous mutations between the two groups of variants. The putatively newly derived mutations are slightly but just significantly (54% as opposed to 51%, p = 0.04 Fisher's exact test) more frequently nonsynonymous, which argues against an extended time of mutation accumulation under conditions of relaxed purifying selection.

#### Recombination happened rather recently

Finally, we asked if the high heterozygosity of Chinese samples is an effect of hybridization events in the relatively recent past. We reconstructed phylogenetic trees of *S*. *stercoralis* males based on the largest autosomal and X chromosomal contigs (S1 Fig). The apparent phylogenetic relationships between the three Chinese samples changed dependent on the genomic region selected, which is clear evidence for recombination in the rather recent past. One possible interpretation is that rare recombination events did occur after the lineage became mostly asexual. Alternatively, the recombined chromosomes might have been present in the population that gave rise to the asexual lineage.

Taken together, it is unlikely that the high heterozygosity in Chinese population was caused by the accumulation of novel mutations after the transition to asexuality. It appears more likely that the mutations arose in sexual populations and the high heterozygosity was caused by one or a few rather recent hybridization events that also rendered the population largely if not completely asexual.

## Discussion

Southern China has a subtropical climate which favors a variety of parasites. The main purpose of this study was the isolation and the genomic/genetic characterization of individual *S*. *stercoralis* and not to conduct a prevalence study or a general parasitological survey. Accordingly, the number of people surveyed was rather limited. Nevertheless, there are a few observations worth mentioning. As expected based on our experience as routine diagnostics provider, the predominant helminths detectable by egg floatation were liver fluke and hookworm. Usually, in the routine parasitological diagnosis, the species of the hookworms is not determined. However, in this study we used molecular diagnostic tools and could show that all hookworms found belonged to the species *N*. *americanus*.

Usually, *S*. *stercoralis* is not detectable by egg floatation. Therefore, we used culturing and Baermann funnels to test the presence of this parasite and to isolate live individual worms, which is not normally done in our diagnostic routine.

Very few studies describing *S*. *stercoralis* prevalences in China were recently published in international journals [78–81]. All such studies we are aware of, were conducted by the same research group and in the Yunnan province, which neighbors the Guangxi province. Our study illustrates that *S*. *stercoralis* is also prevalent in Guangxi, a fact that does not come as a surprise if also clinical case reports (usually only available in Chinese language) are taken into account. As a matter of fact, in a review of such cases between 1973 and 2011[82] more human *S*. *stercoralis* cases in Guangxi than in Yunnan were reported.

Hookworm co-infection with *S*. *stercoralis* are common [31]-[37] and they have similar transmission routes. One could therefore expect, that the dynamics and spreading of these two helminths are similar. This study does not support this conclusion. We found only 2 out of the 7 people infected with *S*. *stercoralis* were also positive for *N*. *americanu*s, which corresponds about to the expectation based on the prevalences (17.3% for hookworms and 7.1% for *S*. *stercoralis*). Also, the hookworms were genetically diverse, while the *S*. *stercoralis* were all very closely related. While the auto infection cycle allows *S*. *stercoralis* to maintain an infection for decades, *N*. *americanus*, in absence of new infection, can only persist for the life time of the individual parasites which is in the order of a few years [25]. Taken together, this suggests that in our study population the transmission rate of hookworms is much higher than the one of *S*. *stercoralis*. The *S*. *stercoralis* positive patients have possibly been infected rather long time ago and maintained the infection through the auto infective cycle.

Contrary to earlier studies [50, 51, 53, 54] we found most *S*. *stercoralis* individuals to be heterozygous for different *SSU* haplotypes. While the *SSU* HVR-I haplotypes had all been described in *S*. *stercoralis* before (although almost exclusively in homozygous state), we identified a novel *SSU* HVR-IV haplotype. These findings are of importance for *SSU*-sequence-based diagnostics and taxonomy of *S*. *stercoralis* and closely related species of *Strongyloides*. The occurrence of *S*. *stercoralis* heterozygous for multiple *SSU* haplotypes may, but not necessarily needs to be, related to our second striking observation, namely the virtual absence of free-living adults. The switch between the clonal direct and the sexual indirect cycle in *Strongyloides* spp. is influenced by the environment, in particular the temperature and the immune statues of hosts, and the genetic background [23]. We cannot completely exclude that at different times of the year, when temperatures are different, in our study area, more sexual animals could be found. However, the climatic conditions in Guangxi are comparable with northern Cambodia where we found numerous free-living adults of both sexes at the same time of the year. We can also not fully exclude that the immune status of the hosts in this study very strongly favored females developing into iL3s. However, our genomic analyses suggest that our study population is largely, if not exclusively asexual. We detected a genome wide heterozygosity, which was even higher than the one described as elevated in the Japanese samples by [54]. These authors attributed the observed heterozygosity to the fact that the worms had accumulated mutations during the asexual reproduction through the auto infective cycle since the infection of the particular host individual. We do not think clonal reproduction only within individual patients could explain our observations. First, in our study the heterozygosity was higher. Second, the worms in the different patients were very closely related. Third, the observed fraction of around 50% nonsynonymous changes is similar to the ones observed in wild populations of the free-living nematode *P*. *pacificus* and *C*. *elegans* and considerably lower than in experimental populations of the same two species, where mutations were allowed to accumulate under minimal purifying selection resulting in around 70% - 75% nonsynonymous mutations in coding regions [83, 84]. This indicates that most variants present in our sample did not arise under conditions of relaxed purifying selection. Nevertheless, we did observe a slightly but significantly higher proportion of nonsynonymous changes among the variants not found in the Cambodian population. Given that presumably only a fraction of these variants were recently introduced to our study population. This observation may be a hint for a limited time period of mutation accumulation with reduced purifying selection. Loss of sexual reproduction has been reported for specific isolates of other species of *Strongyloides*. For *S*. *ratti*, largely asexual populations have been described [24] and in a laboratory strain of *S*. *venezuelensis* (HH1, originally isolated from Okinawa Japan) no males and only very few free-living females were observed under various conditions [85].

We favor the hypothesis that our study population has rather recently, but prior to the infection of the current host individuals, become predominantly if not exclusively asexual as a consequence of one or several hybridization events between sexual populations of *S*. *stercoralis*. This is consistent with the observations of high heterozygosity and the origin of most non-reference variants during a period with purifying selection at a level normal for sexual reproduction. It is important to notice that, if this hypothesis is true, the first generation of hybrids must have been capable of sexual reproduction because the results shown in S1 Fig can only be explained if meiotic recombination occurred at least once after the hybridization event. Asexual genotypes then arose in the next generations due to the particular combination of genetic material derived from the parental lines.

## Supporting information

S1 File Fecal sampling and the prevalence of helminths. The table shows the result of two fecal samplings from human hosts in the villages LA and QX, Guangxi, China. Each row represents one person.
(XLSX)

S2 File Number of hookworms genotyped and the *cox1* haplotypes identified. The table shows the number of hookworms collected, genotyped (*SSU*, ITS, and *cox1*) for each positive host, and number of hookworms of the corresponding *cox1* haplotypes found in each host.
(XLSX)

S3 File Number of *S*. *stercoralis* genotyped and the *cox1* haplotypes identified. The table shows the number of *S*. *stercoralis* collected, genotyped (*SSU* and c*ox1*) for each positive host, and the number of *S*. *stercoralis* of the corresponding *cox1* haplotypes found in each host.
(XLSX)

S4 File *S*. *stercoralis* samples for whole genome analysis. The table shows the sample ID, host ID, country, sex, the mean coverage, the fraction of the genome beyond 15x coverage, the origin of the sequences, and the accession number of individual *S*. *stercoralis* used for the whole genome analysis.
(XLSX)

S5 File Copy of the animal experiment approval. (PDF)

S1 Fig Phylogenies of the largest *S*. *stercoralis* contigs. The upper panel shows neighbor-joining trees for the largest sex chromosomal contigs in the three male samples from China, four high quality samples from Cambodia, and two reference samples. Each contig is at least 100kb large and several hundred variant sites were used to reconstruct individual trees. The lower panel shows the equivalent analysis for autosomal contigs.
(TIF)
