# Supplementary material for: Characterization of a non-sexual population of Strongyloides stercoralis with hybrid 18S rDNA haplotypes in Guangxi, Southern China
Source: PLoS Negl Trop Dis. 2019 May 6;13(5):e0007396. doi: 10.1371/journal.pntd.0007396 (PMC6522072; doi:10.1371/journal.pntd.0007396)
Supplement: S5 File — (PDF) [file pntd.0007396.s005.pdf]

动物实验伦理审查表

The Tab of Animal Experimental Ethical Inspection

编号(No): 20180510

申请人填写的相关信息 (Concerned information written by applicant)

申请人(Applicant)：Dengyu Liu

申请人学历 (Education of applicant)：Master　　技术职称 (Professional title)：Professor

实验名称(Study title)：A non-sexual population of *Strongyloides stercoralis* in Guangxi

实验目的(Aim of experiment)：Culture *Strongyloides stercoralis* in gerbils

基金来源(Fund sources)：Max-Planck-Institute for Developmental Biology

拟进动物情况

动物来源(Source of animal)：Zhejiang Medical College

品种品系(Species or strain)：*Meriones Unguiculatus* (gerbil)

等级(Grade)：SPF　　规格(Specifications)：4-week-old

数量(Number)：2 只(♀ 2 只；♂ 只)　　申请日期(Application date)：2018 年 5 月 10 日

进驻日期(Entering date)：2018 年 5 月 20 日　　结束日期(Ending date)：2018 年 7 月 1 日

1.实验要点，实验方法、观测指标

Outline of experiments； experimental methods；observational index：

Culture *Strongyloides stercoralis* in gerbil: About 300 infective larvae of *Strongyloides stercoralis* are washed and injected subcutaneously at the neck of the gerbil. Feces of gerbil are collected daily for 1 month starting from 7-day post infection. Feces are then moisturized with water and incubate at ambient temperature for 1 day followed by Baermann analysis. *Strongyloides stercoralis* are examined under stereoscope and isolated for molecular and genetic analysis.

2. 仁慈终点或实验终点：

Human endpoint or experimental terminative indicator

Experimental terminative indicator: 37 days after the infection of *Strongyloides stercoralis*.

3.实验结束后处死动物的方法：

Executing animal method：

Gerbils are euthanized with CO_2 after the experiment.

4.动物替代、减少动物用量、降低动物痛苦伤害的主要措施等：

Major measure for 3Rs：

This study design and animal treatment has been refined. Gerbils are the only suitable model for *S. stercoralis* infection except dogs. Minimum 2 animals are needed for this experiment.

申请人签名(Signature of applicant)：[illegible]　　联系电话(Telephone)：13367805975

审查结果 (是否同意

课题负责人意见 (Study director)：　同意 (Agree) ☑　　不同意 (Disagree) ☐　　签名 (Signature) [illegible]

申请人的实验方案)<br>(Results of inspection)	实验动物设施意见(Opinion from laboratory animal facility)：<br><br>同意(Agree) □ 不同意(Disagree) □<br><br>签　　名<br>(Signature)
	实验动物福利与伦理委员会意见(The Animal Care & Welfare Committee)：<br><br>同意(Agree) □ 不同意(Disagree) □<br><br>广西医科大学实验动物伦理委员会<br><br>签　　章<br>(Stamp)
备注(Supplement)：<br>初审 □<br>First trial	第　　次审查<br>reexamine No.

填表须知：动物伦理审查遵循中华人民共和国科技部发布的《关于善待实验动物的指导性意见》及中华人民共和国国家标准 GB/T35892-2018《实验动物 福利伦理审查指南》，如有不明确之处请参看这两份文件或咨询广西医科大学实验动物福利与伦理委员会。

Notes : Animal ethics review follows the *Guiding Opinions on the Treatment of Laboratory Animals* issued by the Ministry of Science and Technology of the People's Republic of China and the *Laboratory Animal-Guideline for Ethical Review of Animal Welfare* issued by the National Standard GB/T35892-2018 of the People's Republic of China. If there are any uncertainties, please refer to these two documents or consult The Animal Care & Welfare Committee of Guangxi Medical University.
