# Supplementary figures and images for: Characterization of a non-sexual population of Strongyloides stercoralis with hybrid 18S rDNA haplotypes in Guangxi, Southern China
Source: PLoS Negl Trop Dis. 2019 May 6;13(5):e0007396. doi: 10.1371/journal.pntd.0007396 (PMC6522072; doi:10.1371/journal.pntd.0007396)

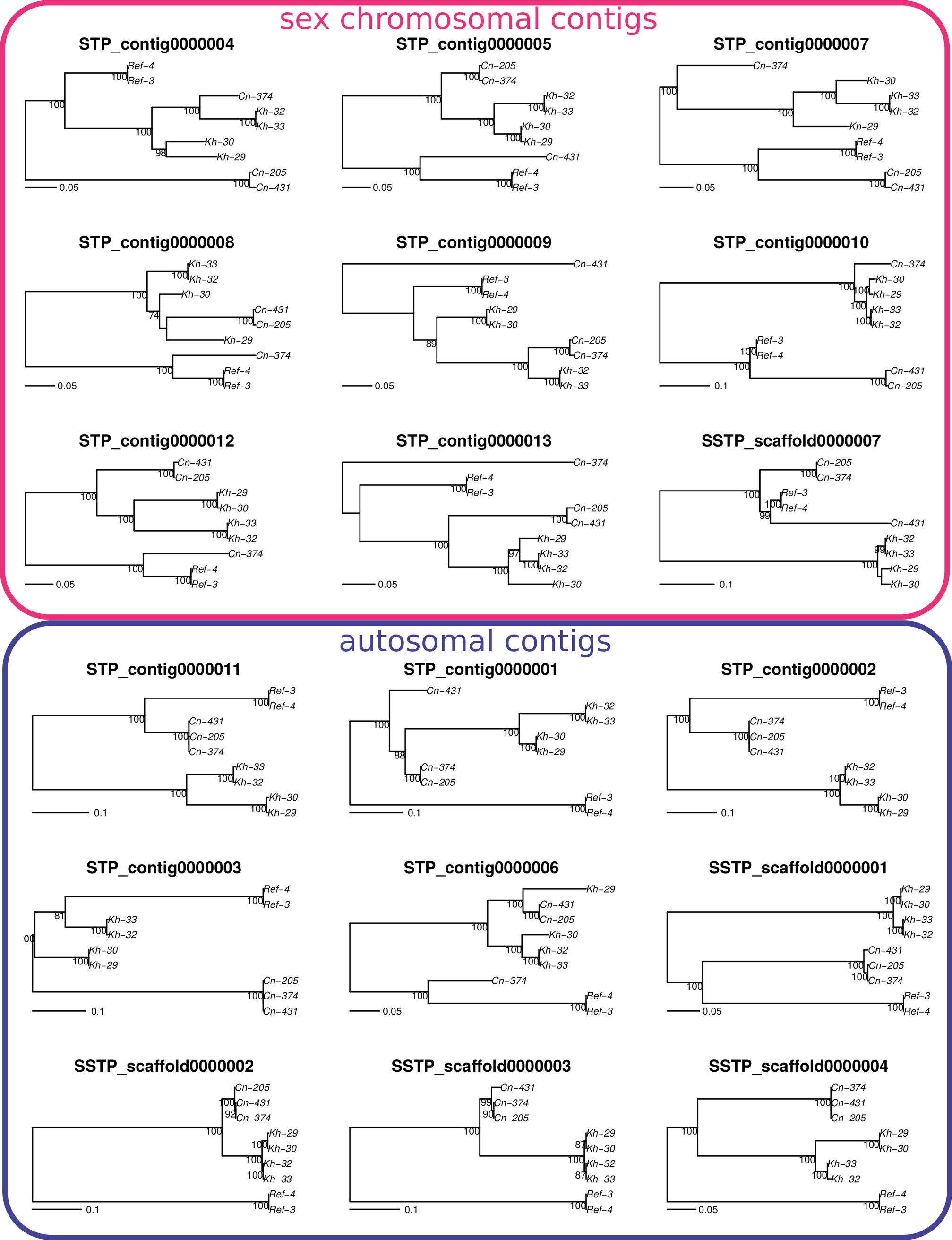

Supplement: S1 Fig — The upper panel shows neighbor-joining trees for the largest sex chromosomal contigs in the three male samples from China, four high quality samples from Cambodia, and two reference samples. Each contig is at least 100kb large and several hundred variant sites were used to reconstruct individual trees. The lower panel shows the equivalent analysis for autosomal contigs. (TIF) [file pntd.0007396.s006.tif]
